# Microdosing Psychedelics to Restore Synaptic Density in Schizophrenia

**DOI:** 10.3390/ijms26188949

**Published:** 2025-09-14

**Authors:** Jacopo Sapienza, Marco Spangaro, Stefano Comai, Michel Sabé, Joseph La Torre, Matteo Buonarroti, Roberto Cavallaro, Marta Bosia

**Affiliations:** 1 IRCCS San Raffaele Scientific Institute, 20127 Milan, Italy; 2 Department of Humanities and Life Sciences, University School for Advanced Studies IUSS, 27100 Pavia, Italy; 3 Department of Pharmaceutical and Pharmacological Sciences, University of Padua, 35123 Padua, Italy; 4 Department of Psychiatry, McGill University, Montreal, QC H3A 0G4, Canada; 5 Department of Biomedical Sciences, University of Padua, 35123 Padua, Italy; 6 Division of Adult Psychiatry, Department of Psychiatry, University Hospitals of Geneva, 1211 Thonex, Switzerland; 7 Faculty of Medicine, University of Geneva, 1211 Geneva, Switzerland; 8 School of Psychology, University of Ottawa, Ottawa, ON K1N 6N5, Canada; 9 Department of Psychiatry and Behavioral Sciences, Center for Novel Therapeutics in Addiction Psychiatry, School of Medicine, University of Washington, Seattle, WA 98104, USA; 10 Società Italiana di Medicina Psichedelica (SIMEPSI), 70124 Bari, Italy; 11 School of Medicine, Vita-Salute San Raffaele University, 20132 Milan, Italy

**Keywords:** LSD, psilocybin, psychosis, negative symptoms, cognition, microglia, complement 4, neuroplasticity, synaptogenesis, SV2A

## Abstract

Schizophrenia is a highly polygenic disease, and several genetic variants associated with the disease converge on altered synaptic homeostasis. In particular, the gene encoding complement component 4 (C4) showed the strongest association with schizophrenia, and this protein is involved in complement-dependent and microglia-mediated synaptic pruning. As a matter of fact, microglia are overactive in schizophrenia, and reduced synaptic arborization, especially in the prefrontal cortex (PFC), is an established hallmark of schizophrenia, likely associated with gray matter loss, cortical thinning, hypofrontality, and deficit syndrome. The recent development of a new radioligand targeting the synaptic vesicle glycoprotein 2A (SV2A) demonstrated in vivo lower synaptic density at the PFC level in individuals with schizophrenia, corroborating the synaptic hypothesis of thedisease first proposed by Feinberg in 1982. Interestingly, robust preclinical evidence (in vitro and animal models) showed the ability of psychedelics to promote neuroplasticity and synaptogenesis, potentially counteracting the excessive synaptic loss, restoring volume loss, and possibly explaining improvements in negative and cognitive symptoms described by old clinical studies. Overall, microdoses should be explored first as a possible treatment in a selected sample of patients affected by deficit schizophrenia, followed by low and full doses if encouraging results were to emerge.

## 1. Introduction

Schizophrenia is a chronic psychotic disorder characterized by high heterogeneity in terms of etiopathology, symptomatology, and clinical course. Symptoms are typically grouped into three main categories: positive, negative, and cognitive symptoms [1]. Positive symptoms usually show a good response to first-line antipsychotics or clozapine in almost 80% of cases, despite often not being complete, with persistence of some residual and subtle symptoms. Negative and cognitive symptoms are typically refractory to available pharmacological treatments and considered unmet clinical needs [2]. Unfortunately, they progressively worsen over time and represent the main determinants of loss of daily functioning and disability [3,4]. Indeed, schizophrenia represents one of the major causes of global disease burden, with important implications in terms of costs for society [5]. Precisely, the economic burden of schizophrenia accounts for around 1.5–3% of healthcare expenditure and 22% of mental health expenses in developed countries. Half of the expenditure is due to direct costs, such as hospitalization, residential facilities, and semi-residential facilities. At the same time, the remaining part is due to indirect costs deriving from functional disability [6]. Then, one of the most significant challenges of our time is to identify possible treatment strategies that can improve symptom dimensions (negative and cognitive symptoms) related to poor functional outcomes, thereby reducing disability [7]. Within the spectrum of the possible clinical manifestations of schizophrenia, deficit-schizophrenia is a particular subtype of illness defined by enduring, primary/idiopathic negative symptoms, thus related to the poorer functional outcome [7]. It is important to stress the concept of “primary”, as these symptoms are not caused by other symptom dimensions, side effects of pharmacotherapy, or intellectual disability [8]. Therefore, they cannot be effectively treated without targeting the underlying biological bases. As a matter of fact, heterogeneity in schizophrenia also concerns biology, as a myriad of systems and pathways are involved in the pathogenesis of the disease [9]. Focusing on negative and cognitive symptoms, the biological underpinnings of “hypofrontality” represent the main determinants of such symptom dimensions [10]. Reduced volumes in frontal and temporal lobes, and particularly in the prefrontal cortex (PFC), are a typical hallmark of schizophrenia, associated with poor cognitive functioning and prominent negative symptoms [10,11,12]. Volume loss can be evident since illness onset [13], and longitudinal studies demonstrated that elevated rates of cortical gray matter loss are associated with conversion from clinical high risk to a psychotic illness [14,15]. Cortical thinning progresses over the course of the disease as both cross-sectional [16] and longitudinal imaging studies suggested that there is an accelerated age-related decline of gray matter volume in schizophrenia patients compared with controls [13,17,18]. Overall, it is likely that neurodevelopmental aspects and neuroinflammation can partially explain volume loss and cortical thinning, and, interestingly, dysfunctional synaptic pruning can be the result of them both [19].

## 2. The Synaptic Hypothesis of Schizophrenia

In 1982, Feinberg stated ‘‘too many, too few, or the wrong synapses are eliminated” in schizophrenia, although he was uncommitted about the precise underlying mechanisms [20]. Then, Hoffman and Dobscha revisited the hypothesis proposing hyperpruning at the prefrontal cortex level and the related hypometabolism in this area as the primary causal event in schizophrenia. The two authors postulated that decreased metabolism in the PFC was mediated by axonal pruning and elimination of axonal collaterals instead of cell death, thus saying that “hypofrontality could occur in the absence of findings indicative of a gross neurodegenerative process” [21]. They also hypothesized that reduced axo-axonic and axo-dendritic synapses underlie disrupted interneuron connectivity, functional fragmentation of cortical networks, and genesis of symptoms [21], laying the foundations for the disconnection hypothesis of schizophrenia [22,23,24]. In 1994, Keshavan and colleagues further revisited the synaptic hypothesis, updating it with new findings from post-mortem studies that showed reduced brain volume, cortical thinning, and sulcal enlargement, as well as early MRI studies that revealed reduced frontal lobe volume and reduced cortical gray matter volume [25]. Moreover, the authors also included emerging evidence for frontal hypometabolism inferred from positron emission tomography (PET), magnetic resonance spectroscopy, and fMRI studies [25]. The concept of schizophrenia as a dendritic spine pathology is regaining attention due to recent PET studies targeting the synaptic vesicle glycoprotein 2A (SV2A), a marker of synaptic density, and a revamped interest in the synaptic hypothesis of schizophrenia is occurring [26,27,28]. Indeed, despite the novelty and plausibility of the synaptic hypothesis of schizophrenia, an important limitation was the lack of evidence for an in vivo synaptic deficiency [27]. This limitation has been overcome by the development of a new PET radioligand [11C] UCB-J, specific for the synaptic vesicle glycoprotein 2A (SV2A) expressed by presynaptic terminals, enabling the assessment of synaptic density in living human brains [29,30]. Thus, for the first time, [11C] UCB-J PET studies demonstrated a reduced synaptic density in vivo and particularly in frontotemporal areas [28,29]. Despite the interindividual variability, decreased synaptic density seems to correlate with the duration of illness [28], although it is also detectable in the early course of the disease [31]. These findings are compatible with both the perspectives of schizophrenia conceived as a neurodevelopmental disease and the progressive worsening of negative symptoms and impairment of cognitive functions over the course of illness [15]. Indeed, there is a substantial overlap between frontotemporal areas burdened by cortical thinning and hypometabolism largely described in schizophrenia and frontotemporal areas found to be deprived of synapses [17,27,32]. Considering that dendrites and axons represent 30% and 29% of cortical volume, respectively, it is likely that the progressive volume loss underlying negative and cognitive symptoms is largely due to hyperpruning [17,32]. In this view, parallelisms can be drawn between dementia, caused by neurodegeneration and neuron loss, and schizophrenia (dementia praecox), in which synapses are pruned but neurons’ somas are preserved.

### 2.1. Can Synaptic Loss Explain Cortical Thinning in Schizophrenia?

Kassem and colleagues, by combining structural MRI and confocal microscopy, demonstrated that stressed mice showed gray matter losses of 10 and 15% in the anterior cingulate cortex (ACC) and hippocampus, coupled with a loss of synaptic spine density of up to 60% but no changes in the number or volumes of the somas of neurons, astrocytes, or oligodendrocytes [33]. Moreover, there was a strong linear relationship between dendritic volume loss and MRI-estimated gray matter volume loss [33]. A similar technique was used by Keifer and colleagues in a study on the auditory fear-conditioning paradigm in mice to investigate its effects on different cortical areas. They found increased gray matter voxel intensity in several brain regions compared to the controls. Focusing on the auditory cortex, they described concurrent increases in dendritic spine density with a positive correlation between dendritic spine density and gray matter voxel intensity [34]. Taken together, these preclinical findings indicate that synaptic changes could contribute at least partially to cortical thinning and neurostructural alterations seen in schizophrenia, despite not proving such a relationship.

### 2.2. Genome-Wide Association Studies

The genetic burden underlying schizophrenia can be extremely heterogeneous, as an increasing number of genes have been associated with the disease by genome-wide association studies (GWAS) [35,36,37], with one of the latest and largest GWAS finding 287 associated loci [37]. Among them, several schizophrenia risk variants converge on biological pathways regulating synaptic elimination, formation/organization/differentiation, transmission, signaling, and plasticity, including complement factors and microglial-mediated synaptic pruning [35,37,38]. Another important GWAS found that the gene encoding complement component 4 (C4), a protein involved in the intricate complement system of the major histocompatibility complex (HLA), showed the strongest association with schizophrenia [35,38]. Interestingly, C4 protein is largely involved in microglia-mediated synaptic pruning [38,39].

### 2.3. Complement-Dependent Pruning

**Figure 1:**
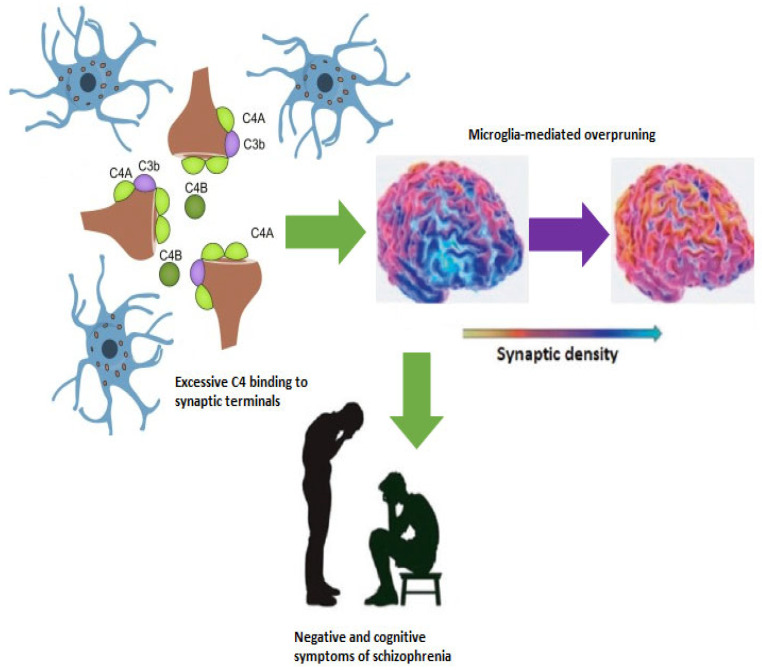
Excessive synaptic pruning reduces synaptic density in the prefrontal cortex with implications for negative and cognitive symptoms.

Several lines of evidence ranging from post-mortem to in vivo PET studies targeting the translocator protein (TSPO), overexpressed after microglial activation in the inflammatory-phenotype M1, have demonstrated the primary role of microglia in determining neuroinflammation in schizophrenia [40,41]. Microglia play a pivotal role in the synaptic complement-mediated pruning in the Central Nervous System (CNS) as demonstrated by the positive correlation between genetically predicted C4 expression and TSPO brain levels [42] and increased elimination of synapses in patient-derived neural cultures and isolated synaptosomes [43]. Notably, increased neuronal C4 deposition and synapse uptake were both associated with risk-associated variants within the human C4 genetic locus [43]. Similar findings were also reported in animal studies, as mice overexpressing C4 showed reduced cortical synaptic density, increased microglial engulfment of synapses, and altered behaviors [39]. Moreover, increased expression of C4 leads to decreased spine density and hypoconnectivity in the PFC, decreased intrinsic excitability, working memory impairment, and reduced social interactions between mice [44,45]. Interestingly, a study by Gangadin and colleagues found that serum levels of C4 protein were negatively related to frontal brain volumes in patients with schizophrenia-spectrum disorders but not in healthy controls, supporting the role of complement-mediated pruning in PFC volume reduction in schizophrenia [46]. Similarly, negative correlations were found between the TSPO signal, an index of microglial activation, and total cortical gray matter volumes in individuals with schizophrenia [47]. Moreover, genetically predicted C4 expression levels were reported to be negatively associated with cortical thickness in several brain regions implicated in schizophrenia in a large UK Biobank sample of over 27,000 subjects, which excluded individuals with diagnoses of neurological or mental disorders [48]. In addition, higher predicted C4 expression levels were negatively associated with middle temporal cortex activation in healthy controls during an fMRI visual processing task, and with episodic memory performance in healthy controls and patients with schizophrenia [49]. Overall, all these findings point to a possible association between increased synaptic pruning, thus decreased synaptic density, PFC volume depletion, and negative and cognitive symptoms in schizophrenia, as shown in Figure 1.

## 3. Psychedelics Promote Neuroplasticity

Classic or serotonergic psychedelics are terms generally used to indicate psychoactive substances able to induce altered perception, typically visual hallucinations or illusions, and an altered state of consciousness [50]. Most of them are semi-synthetics or derived from plants. The class includes the semisynthetic ergoline Lysergic acid diethylamide (LSD), plant-derived tryptamines like psilocybin (the active compound of magic mushrooms) and N,N-dimethyltryptamine (DMT, the active ingredient in ayahuasca), and phenethylamines, such as mescaline (the active compound of the peyote and San Pedro cacti) and many other phenethylamine-based synthetic designer drugs [51,52]. Pharmacodynamics of psychedelics can differ depending on the specific compound; however, all of them exert their pharmacological effects primarily through the serotonergic system, acting as agonists or partial agonists at the 5-HT2A receptor level [50,53]. Along with glutamatergic dissociatives like ketamine and phencyclidine (PCP), which act as NMDA glutamatergic receptor antagonists, psychedelics pertain to the wider class of psychoplastogens [54]. Psychoplastogens, despite the different pharmacodynamic properties, converge on the intracellular pathways of mTOR and TrKB, deputed to the activation of protein synthesis and the main determinants of enhanced neuroplasticity [55,56]. Psychoplastogens are defined as fast-acting compounds capable of inducing a measurable change in terms of neuroplasticity (number of dendrites, spine density, intrinsic excitability) within 24–72 h, following a single administration, and these effects must persist beyond acute stimulation of the substance [54]. Indeed, in vitro studies on cultured neurons exposed to different types of psychedelics showed a different extent of dendrites and neurites proliferation, increased spine density, and synaptogenesis depending on the specific compound used [57,58,59]. According to the definition of psychoplastogens [54], transient stimulation (<1 h) was sufficient to induce neuroplasticity in cultured neurons, which persisted for more than three days [58]. The same findings were replicated in vivo by Shao and colleagues, who used two-photon microscopy to longitudinally assess dendritic spines of layer V pyramidal neurons in brain slices of mouse medial frontal cortex. They found that a single dose of psilocybin led to 10% increases in spine size and density within 24 h from administration and that these changes persisted one month later [60]. Unfortunately, there are still no [11C] UCB-J PET studies able to clarify the potential of psychedelic compounds to increase synaptic density in human brains [61]. However, given the evidence that comes from in vitro and animal studies [57,60] and preclinical molecular findings on increased expression of SV2A and several other synaptic proteins in mouse brains [62], it is likely that such modifications would also occur in living human brains. Finally, it must be acknowledged that in vitro studies showed that newborn prolongations lack both internal and external organization, as they are characterized by an immature elongated cyto-architecture (filopodium-like and not the typical mature mushroom-like spine) and show no organization in the inter-neuronal space [57]. Overall, a revamped interest in psychedelic research spread among psychiatrists in the last decade, and mounting evidence has shown therapeutic properties of psychedelics in many psychiatric diseases, such as anxiety, particularly in the context of life-threatening diseases, substance use disorders, eating disorders, obsessive–compulsive disorder, and neurocognitive disorders. Still, the most promising results have been obtained with psilocybin-assisted therapy for Treatment-Resistant Depression [51]. Indeed, antidepressant properties of psilocybin are widely reported by several randomized clinical trials (RCTs) [63,64,65].

## 4. Anti-Inflammatory Properties of Psychedelics

Psychedelics display anti-inflammatory properties as demonstrated by cell and animal models of inflammation [52]. Specifically, the downregulation of intracellular NFkB pro-inflammatory signaling and TNF-α production seems to be mediated by 5HT2A stimulation [66,67,68]. Activation of 5HT2A receptors modulates the production of pro-inflammatory cytokines and regulates microglial activity [69]. It is likely that mechanisms underlying the anti-inflammatory effects of psychedelics rely on the modulation of histone acetylation/deacetylation state and DNA methylation of genes involved in immune response [70,71]. Indeed, under the effect of psychedelics, genes encoding for vascular cell adhesion molecule-1 (VCAM-1), intracellular adhesion molecule-1 (ICAM-1), and IL-6 were less expressed [68], and decreased IL-1, IL-6, IL-8, and TNF-α, and increased IL-10 were found in activated human monocyte-derived dendritic cells [72]. In addition to direct cytokine modulation, psychedelics may influence inflammation through the kynurenine pathway (KP), the main route of tryptophan catabolism, which generates immunoactive metabolites with neuroprotective or neurotoxic potential [73]. Although the literature on this topic remains limited, as recently reported in the review by Campanale and colleagues [74], psychedelics appear capable of modulating the KP, possibly via effects on key enzymes or through the aryl hydrocarbon receptor (AHR). Notably, both endogenous and exogenous psychedelics can act as AHR ligands, a transcription factor involved in immune regulation, gut barrier integrity, and microglial function.

## 5. Could Psychedelics Be Used to Treat Schizophrenia?

**Figure 2:**
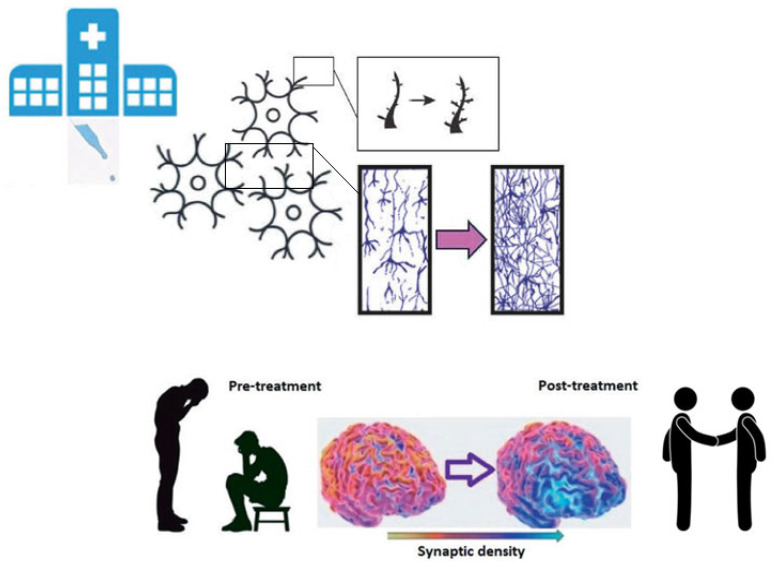
Psychedelic-induced neuroplasticity could restore synaptic density in the prefrontal cortex with positive implications for symptoms.

Until recently, individuals with psychotic disorders were largely excluded from psychedelic research [75,76]. However, a growing body of scholars now advocates for exploring potential therapeutic benefits in this population, as mounting evidence is pointing to a relative safety of psychedelic use by patients with psychotic disorders, as demonstrated by extensive longitudinal studies [77,78,79] and meta-analytic evidence [80], but also suggested by old clinical studies [7]. However, most of such evidence is based on self-reports and not confirmed by recent clinical studies, of which there is an urgent need. Overall, this perspective paper focuses on the possibility of using psychedelics to treat schizophrenia in a strict biological and neuroscientific framework rather than broadly answering the question “Can psychedelics treat schizophrenia?”. Among all the possible therapeutic mechanisms, the anti-inflammatory effect at the central level and the putative ability to restore synaptic density in the PFC are two of the most plausible and fascinating. Several meta-analyses showed increased levels of pro-inflammatory cytokines in individuals with schizophrenia, and particularly TNF-α, IL-1β, and IL-6 [81,82,83]. Neuroinflammation plays a pivotal role in the pathogenesis of schizophrenia [19,84] and, from the perspective of schizophrenia, conceived as a synaptic pathology, microglial activation and complement-mediated synaptic pruning are the most important mechanisms [35,39]. Increased synaptic pruning, triggered by neuroinflammation, represents one of the most important pathogenetic mechanisms of schizophrenia, likely responsible for most of the progressive cortical thinning at the PFC level and related cognitive and negative symptoms [19,27,32]. Therefore, given the ability of psychedelics to reduce immune activation in the CNS and their primary synaptogenic properties, they could be a valuable treatment option to restore synaptic density and volume loss in the PFC of individuals with schizophrenia. On the one hand, psychedelics could probably resize pruning extent through their anti-inflammatory effect, and, on the other hand, enhance synaptogenesis. Hypothetically, these mechanisms could induce cortical thickening, associated in turn with improvements in negative and cognitive symptoms, especially if coupled with rehabilitation strategies like cognitive remediation therapy, as shown in Figure 2. Supporting evidence on the possible role of psychedelics in the treatment of negative symptoms and socio-cognitive deficits was provided by old past-century pioneering studies on the administration of psychedelics to patients with schizophrenia [7]. Increased number of social interactions, enhanced talkativeness, and more expressed affection were reported along with improved understanding of social situations and environmental stimuli in daily life situations and more appropriate reactions to them [85,86,87,88,89,90]. Notably, the number of social interactions increased at lower LSD dosages (25–50 µg) and decreased at higher doses (100–200 µg) [88]. In accordance with these clinical findings, psychedelics showed the ability to reopen the social reward learning period in rodents with positive implications for social behaviors [91]. In addition, another study proved that LSD-induced social interactions among mice were mediated by mTOR signaling, which is involved in protein synthesis and underlies neuroplasticity [92]; thus, increased social interactions are likely related to synaptogenesis. Given the evidence of the potential that psychedelics could have in the treatment of schizophrenia with remarkable implications for the clinical outcome for patients, Tuck and colleagues recently synthesized a structural analog of LSD with lower hallucinogenic potential, proving its unaltered potent neuroplasticity-promoting properties to overcome contraindication for schizophrenia [93]. Such a way of conceiving the therapeutic benefits of psychedelics (enhanced neuroplasticity), achievable without hallucinogenic effects, needs to be endorsed by further evidence. Moreover, this perspective emphasizes perceived clinical risks of sub-hallucinogenic experiences that may not affect the clinical outcome of psychotic patients.

### 5.1. Safety and Feasibility

These findings suggest that the use of psychedelics for the treatment of schizophrenia would be based on a strong biological rationale, but the administration of psychedelics to patients with schizophrenia represents a controversial issue, as psychedelics are psychotropic substances historically associated with exacerbation or onset of positive symptoms, and their pharmacodynamics were used to recreate drug models of the disease [94,95]. However, psychedelic-induced psychosis and positive symptoms of schizophrenia are very different, as psychedelics produce transient complex visual hallucinations (complex imagery, Kaleidoscope), ego dissolution, disembodiment, and audio-visual synesthesia [96,97] at relatively high doses (LSD > 100 µg per os), and acute intoxication can be avoided by using low doses [98]. On the other hand, positive symptoms of schizophrenia typically are represented by auditory hallucinations and persecutory or other types of delusions [1]. As a matter of fact, recent meta-analytic findings underscore the need to reconsider schizophrenia as an inclusion criterion for clinical trials exploring the safety and efficacy of psychedelics, as the incidence of psychedelic-induced psychosis has been 0.002% in population studies, 0.2% in uncontrolled trials (UCTs), and 0.6% in randomized-controlled trials (RCTs). 3.8% of individuals with schizophrenia developed long-lasting psychotic symptoms in UCTs, and of those with psychedelic-induced psychosis, 13.1% later developed schizophrenia [80]. Considering past-century studies on the administration of psychedelics to patients with schizophrenia, some reports described increased anxiety, agitation, and worsening of positive symptoms [99,100,101] but with doses of LSD > 100 µg. Differently, Bender and colleagues reported no “acute psychotic reaction” even if patients were treated with doses up to LSD 150 µg [86]. Similar findings were reported by other authors who acknowledged that psychotic patients were very resistant to the effects of LSD (>200 µg os or >100 µg intramuscular) and higher doses of LSD were needed to exert symptoms of acute intoxication in psychotic patients relative to healthy subjects [100,102,103,104]. In addition, Paul Hoch in 1955 stated that chronic and deteriorated patients with schizophrenia showed very little response to LSD and mescaline compared to “acute schizophrenic patients” who responded with great intensity [105]. Thus, clinical high-risk individuals and first-episode patients should not be included in future clinical trials, but only patients with schizophrenia showing treatment-resistant negative, cognitive, and depressive symptoms. It is important to underscore the dimension of depressive symptoms in schizophrenia, often entangled with negative symptoms, particularly in the long-term course of the disease, and often not adequately considered by clinicians [106,107]. 70% of patients with schizophrenia show a lifetime incidence of depressive symptoms, and 30% of them present clinical comorbid depression [107]. Psychedelics may also reduce this component, as their antidepressant effect has been demonstrated by several clinical trials, particularly on treatment-resistant depression.

#### Microdosing

Full doses of psychedelics undoubtedly induce symptoms related to acute intoxication, and, even if individuals with schizophrenia showed good resistance to them, they should be avoided in future clinical trials. A possible option to minimize risks could be microdosing, the practice of taking 10% of the pharmacologically active dose to not reach the “psychedelic threshold”, with only modest or no psychoactive effects [108]. The importance of micro or small doses was underscored by Cheek and Holstein, who stated that the number of social interactions increased with LSD 25–50 µg but decreased with 100–200 µg [88]. Similar findings were also reported by Busch and Johnson, who found positive effects on negative symptoms with LSD 30–40 µg [89]. Regarding concerns about the potential risk of microdosing-induced valvulopathy, stemming from the affinity of classical psychedelics for the 5-HT2B receptor, recent evidence appears to rebut this correlation, thereby suggesting a more favorable safety profile [109].

### 5.2. Possible Target Population and Treatment Protocol

Patients affected by deficit-schizophrenia, or comorbid depression or depressive symptoms, or in a stable, residual phase of illness characterized by more prominent negative and cognitive symptoms, should be the target population for future pilot studies or clinical trials [7]. Microdoses or low doses of psychedelics, preferably psilocybin, given higher safety and manageability compared to LSD, should be associated with ongoing antipsychotic treatments to minimize the risk of psychotic exacerbation and administered two or three times a week in a day-hospital regimen and under medical supervision [7]. Two/three times per week should be appropriate due to the long-lasting effect in terms of induced neuroplasticity [54]. The association with antipsychotics, even if second-generation antipsychotics, should not hamper the neuroplastic effect, given that antipsychotics act on 5-HT2A receptors on the neuron surface, while there is evidence that psychedelics elicit their action through an intracellular pool of 5-HT2A receptors according to molecular findings by Vargas and colleagues [53]. This explains why polar compounds that bind external 5-HT2A receptors (serotonin) do not elicit the same effect, because they are unable to cross cellular membrane [53,110]. Therefore, psychedelic compounds and second-generation antipsychotics blocking 5-HT2A receptors could act at different pharmacodynamic levels. Nonetheless, there is evidence that risperidone and olanzapine can attenuate—or even block—the psychedelic experience [111,112]. This could mainly be due to a shared action on receptors located on the neuron surface or to a milder lipophilic activity of second-generation antipsychotics. An important matter of debate is whether 5-HT2A–mGlu2 co-activation in humans is strictly required for the therapeutic effects of psychedelics [113,114]. If this turns out to be true, it could be a further mechanism of differentiation at the pharmacodynamic level between the action of psychedelics and antipsychotic drugs. Anyway, what does appear to persist, in any case, is the ability to activate the TrkB intracellular pathway, different from antipsychotics [115]. Consequently, it could be hypothesized that prioritizing antipsychotics with stronger D2/D3 and less 5HT2A blockade in early future clinical trials would reduce confounding variables.

## 6. Future Directions

There is an urgent need to assess if psychedelic-induced synaptogenesis is a mechanism occurring also in living human brains and not only in vitro or in animal models. Moreover, the in vivo potential of microdoses to induce synaptogenesis should be assessed as well in future studies employing the [11C] UCB-J PET technique. Future studies should also test the hypothesis of a maintained ability to promote neuroplasticity of psychedelics, even if combined with concomitant antipsychotic treatments, or find the most suitable antipsychotic for the combination. Furthermore, microdoses may not be sufficient to fully restore synaptic density and cortical thickness; thus, testing higher doses may be necessary in future studies in an attempt to find the lower effective dose. Even the ability of non-hallucinogenic analogs to induce synaptogenesis should be tested in vivo, possibly identifying a valuable molecule for the treatment of schizophrenia. Pilot studies on small samples of patients with deficit or chronic schizophrenia should be performed to test safety and feasibility. Then, RCTs on larger samples to test differences in terms of outcome between the drug-placebo arms should be performed. Within the context of clinical studies, [11C] UCB-J PET longitudinal assessments should be integrated in the study protocol to investigate the putative association of clinical improvements (negative and cognitive symptoms) with increased synaptic density and, hopefully, cortical thickening at the PFC level. Finally, studies comparing the association of rehabilitative strategies combined with psychedelic treatment versus only psychedelic treatment should take place to test the hypothesized further beneficial effects.

## 7. Conclusions

Mounting evidence suggests that psychedelics may hold therapeutic potential for the negative and cognitive symptoms of schizophrenia, two symptom dimensions typically resistant to current available treatments. Their ability to promote synaptic remodeling in the PFC is particularly relevant, given the role of aberrant synaptic pruning in the pathophysiology of schizophrenia. From a conservative and safety-focused perspective, we propose that carefully designed clinical studies exploring microdosing in a well-defined subgroup of patients with prominent negative and cognitive symptoms could be justified, particularly considering the current lack of effective therapeutic alternatives. Overall, the potential benefits in terms of improved functional outcomes and quality of life may outweigh the risks of exacerbating positive symptoms, especially when appropriate screening and monitoring are in place.

## Data Availability

Not applicable.
